# Isolated Extra-articular Medial Dislocation of the Long Head of the Biceps Tendon in an Adolescent: A Case Report

**DOI:** 10.7759/cureus.102376

**Published:** 2026-01-27

**Authors:** Job Alejandro Reyes Jasso, A Lezith Marroquin Rodriguez, Yendi Fernanda Ayala Pérez, Francisco Rafael Espinosa Leal

**Affiliations:** 1 Radiology, Hospital Regional del ISSSTE Monterrey, Monterrey, MEX; 2 Radiology, Hospital Regional de Alta Especialidad de la Península de Yucatán, Mérida, MEX; 3 Radiology, Hospital Regiomontano, Monterrey, MEX

**Keywords:** adolescent shoulder pain, biceps tendon instability, long head of the biceps tendon, mr arthrography, shoulder mri

## Abstract

Isolated instability of the long head of the biceps tendon is uncommon and is usually associated with rotator cuff or bicipital pulley injuries. Isolated extra-articular medial dislocation in the absence of associated lesions is particularly rare in the adolescent population. We report the case of a 15-year-old male who presented with anterior shoulder pain after a recreational sports activity. Physical examination revealed localized anterior and anterolateral tenderness, with preserved active and passive range of motion and no clinical signs of instability. Magnetic resonance imaging using MR arthrography demonstrated an isolated extra-articular medial dislocation of the long head of the biceps tendon, characterized by an empty bicipital groove and preserved integrity of the rotator cuff, rotator interval, and labro-bicipital complex. Conservative management with physical therapy focused on pain control and shoulder stabilization was initiated, along with activity modification and injury prevention counseling. The patient showed a favorable clinical course during conservative management. This report highlights the importance of systematic evaluation of the bicipital groove and the entire course of the long head of the biceps tendon on MRI in young patients with anterior shoulder pain, even when classically associated injuries are not identified.

## Introduction

The bicipital pulley system, formed primarily by the superior glenohumeral and coracohumeral ligaments and reinforced by adjacent fibers of the subscapularis and supraspinatus tendons, plays a key role in the stabilization of the long head of the biceps tendon as it exits the glenohumeral joint through the rotator interval [1-4]. Under normal conditions, the long head of the biceps tendon originates from the supraglenoid tubercle or superior labrum, courses intra-articularly, and then enters the bicipital groove. Medial displacement is biomechanically abnormal and is typically associated with injury to the stabilizing pulley structures [1,5-7]. Instability of the long head of the biceps tendon is uncommon, and medial subluxation or dislocation is most often described in adults with degenerative or traumatic rotator cuff pathology, frequently involving disruption of the pulley complex and or subscapularis tendon abnormalities [1,2,5-8]. In contrast, its occurrence in adolescents is exceptionally rare and has been reported only sporadically in isolated cases [8-11].

The gap in the literature becomes more evident when considering that most reported adolescent cases describe associated structural abnormalities of the subscapularis or the pulley system. The present report is unusual because it demonstrates an isolated extra-articular medial dislocation of the long head of the biceps tendon, with preserved integrity of the rotator cuff, rotator interval, and labro-bicipital complex on MR arthrography. Clinically, this entity may present with nonspecific anterior shoulder pain and preservation of range of motion, making imaging essential for diagnosis and for the exclusion of associated injuries [5-8]. In adolescents, acute rotational stress during recreational sports such as arm wrestling or repetitive overhead activity may contribute to instability even in the absence of obvious structural damage [9-13].

## Case presentation

A 15-year-old male patient with no relevant medical history presented with anterior right shoulder pain. One year before the consultation, he had experienced an episode of acute shoulder pain during a recreational arm wrestling match. The pain resolved spontaneously, and no medical evaluation or treatment was sought at that time. The patient remained asymptomatic for several months following the incident. In recent months, after beginning to play paddle tennis occasionally and recreationally, involving repetitive overhead and rotational shoulder movements, he had developed sudden-onset sharp pain in the anterolateral aspect of the right shoulder during play. The pain occurred without direct trauma or radiation.

**Figure 1:**
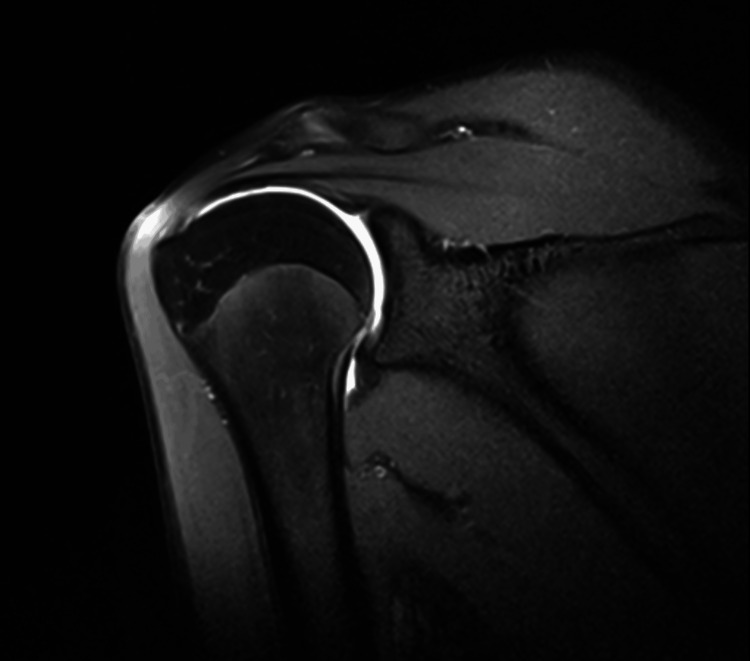
Coronal T1-weighted fat-suppressed MR arthrography image of the right shoulder demonstrating intra-articular contrast distribution with adequate capsular distension

On physical examination, the anterior and anterolateral areas of the right shoulder were tender to palpation. There were no visible deformities or swelling, and no clinical signs of instability were observed. No neurovascular deficits were identified, and both active and passive ranges of motion remained intact. Given the nonspecific clinical presentation and the need to optimize evaluation of the labro-bicipital complex, rotator interval, and subtle patterns of instability, magnetic resonance imaging was performed as an MR arthrogram. The study employed spin-echo techniques, including T1-, T2-, and inversion recovery sequences acquired in axial, sagittal, and coronal planes, both before and after intra-articular contrast administration (Figure 1).

**Figure 2:**
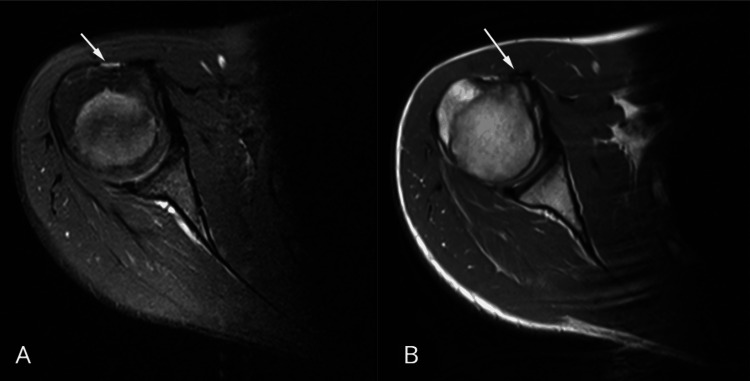
Axial MRI images of the right shoulder A: proton density fat-suppressed sequence demonstrating an empty bicipital groove (arrow). B: axial T2-weighted fat-suppressed MR arthrogram demonstrates medial extra-articular displacement of the long head of the biceps tendon (LHBT) relative to the bicipital groove, with preservation of the normal morphology of the subscapularis tendon (arrow)

Adequate capsular distension was achieved after contrast injection, allowing optimal evaluation of the labro-ligamentous structures. Imaging demonstrated extra-articular medial dislocation of the long head of the biceps tendon, superficial to the subscapularis tendon, with an empty bicipital groove (Figure 2).

**Figure 3:**
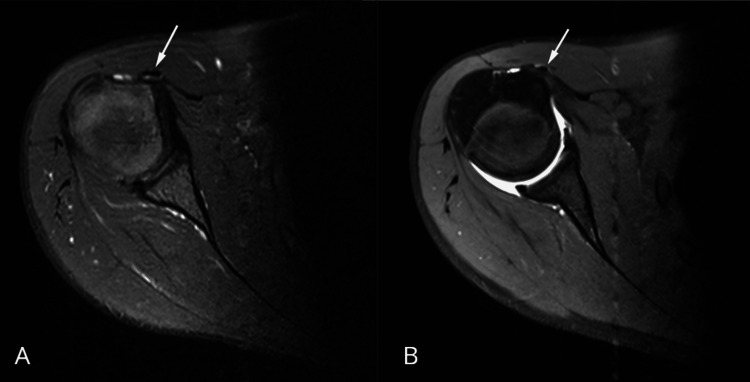
Axial MRI images of the right shoulder A: proton density fat-suppressed sequence demonstrating medial displacement of the long head of the biceps tendon outside the bicipital groove (arrow). B: axial T1-weighted fat-suppressed MR arthrography image with intra-articular contrast confirming extra-articular medial dislocation of the long head of the biceps tendon (arrow)

Along its intra-articular course, the tendon appeared thinned from its origin at the supraglenoid tubercle and superior labrum, without discontinuity or abnormal signal intensity suggestive of tearing. Axial proton density fat-suppressed and axial T1-weighted fat-suppressed MR arthrography images confirmed medial displacement of the tendon and its extra-articular position (Figure 3).

**Figure 4:**
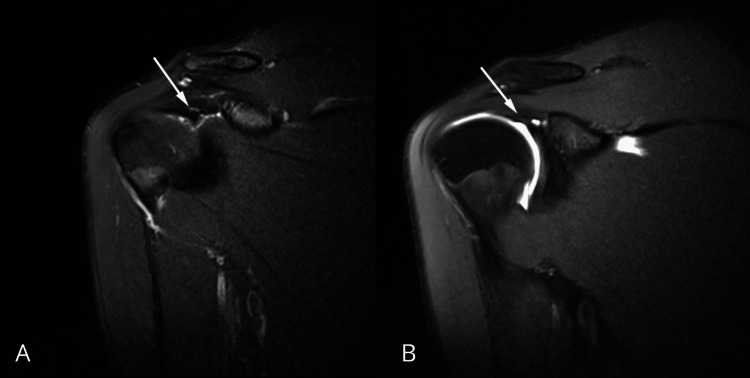
Coronal MRI images of the right shoulder A: proton density fat-suppressed sequence showing preserved integrity of the coracohumeral ligament and normal rotator interval anatomy. B: T1-weighted fat-suppressed MR arthrography image with intra-articular contrast confirming intact coracohumeral ligament, without evidence of rotator interval or bicipital pulley system injury

**Figure 5:**
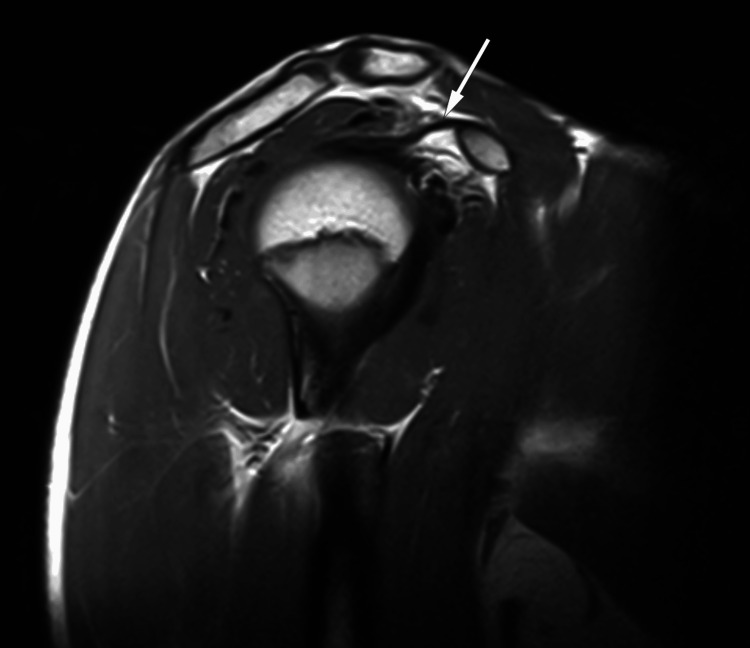
Sagittal proton density-weighted MRI of the right shoulder Demonstrating preserved integrity of the coracohumeral ligament, with normal appearance of the rotator interval and no evidence of bicipital pulley system injury

The rotator interval showed normal morphology and signal intensity. The rotator cuff tendons, including the subscapularis, supraspinatus, infraspinatus, and teres minor, demonstrated preserved structural integrity, with no evidence of tendinosis or partial- or full-thickness tears. The coracohumeral ligament demonstrated preserved integrity, without imaging evidence of rotator interval or bicipital pulley disruption (Figures 4, 5).

The glenoid labrum and glenohumeral ligaments were normal, with a sublabral recess identified as a normal anatomic variant. Bone marrow signal intensity was normal, without evidence of edema or osseous injury.

Based on the imaging findings, a diagnosis of isolated extra-articular medial dislocation of the long head of the biceps tendon was established. Conservative management with physical therapy focused on pain control and shoulder stabilization was initiated, along with recommendations regarding physical activity modification and injury prevention. The patient demonstrated favorable clinical evolution during follow-up under conservative management.

## Discussion

Isolated instability of the long head of the biceps tendon is rare, as medial subluxation or dislocation is most commonly associated with tears of the subscapularis tendon and/or disruption of the biceps pulley system [1,2,5]. Most descriptions involve adult patients with rotator cuff pathology, whereas isolated medial dislocation without concurrent rotator cuff injury has been reported only in a limited number of cases [3,4]. The present case is unusual because an extra-articular medial dislocation occurred in an adolescent patient without detectable structural injury on MR arthrography to the rotator cuff, rotator interval, or labro-bicipital complex. Similar rare cases with intact rotator cuff tendons have been described in the literature, challenging the traditional notion that subscapularis disruption is required for biceps tendon instability [2,3]. Rather than reiterating the imaging findings, the main contribution of this report is to emphasize the importance of careful, systematic assessment of the bicipital groove and the entire course of the long head of the biceps tendon on MRI in young patients with anterior shoulder pain, even when classic associated injuries are absent [3,4,12].

The stabilizing function of the biceps pulley system, composed primarily of the coracohumeral and superior glenohumeral ligaments, has been described in anatomic and imaging studies [1,5,6]. However, MRI primarily assesses structural integrity rather than dynamic or functional competence. One possible explanation for isolated instability in the absence of structural disruption, particularly in adolescents, is transient, microstructural, or functional insufficiency of pulley components; this interpretation should be considered speculative. In this context, subtle microstructural damage or dynamic instability cannot be fully excluded and represents a limitation of static imaging techniques [4,12].

Sports-related mechanisms involving rotational and eccentric loading of the shoulder, such as arm wrestling and repetitive overhead activities, have been linked to a variety of shoulder injuries even without direct trauma [11-13]. In the present case, the initial arm wrestling episode may have predisposed the tendon to instability during subsequent overhead activity. From a clinical perspective, the choice between conservative and surgical management depends on symptoms, associated injuries, and functional impact. In selected patients without associated structural damage, conservative management may be an appropriate initial approach, as demonstrated in this case. The main limitation of this report is its single-case nature, which limits generalization of diagnostic or management recommendations.

## Conclusions

Isolated extra-articular medial dislocation of the long head of the biceps tendon is a rare entity, particularly in adolescent patients. This report highlights the importance of a systematic evaluation of the bicipital groove and the entire course of the long head of the biceps tendon on MRI in young patients presenting with anterior shoulder pain, even when associated rotator cuff or pulley injuries are absent. In selected adolescent patients without demonstrable structural damage, conservative management may be an appropriate initial approach. Given the single-case nature of this report, clinical inferences and management recommendations should be interpreted with caution.

## References

[1] Walch G, Nové-Josserand L, Boileau P, Levigne C (1998). Subluxations and dislocations of the tendon of the long head of the biceps. J Shoulder Elbow Surg.

[2] Gambill ML, Mologne TS, Provencher MT (2006). Dislocation of the long head of the biceps tendon with intact subscapularis and supraspinatus tendons. J Shoulder Elbow Surg.

[3] Cho CH, Lee KJ, Bae KC (2008). Isolated medial dislocation of the long head of the biceps without rotator cuff tear: a case report. J Korean Orthop Assoc.

[4] Vopat ML, Yang SY, Gregor CM, Kallail KJ, Saunders BM (2020). Medial dislocation of the long head of the biceps without concomitant subscapularis tear: a case report. J Orthop Case Rep.

[5] Lafosse L, Reiland Y, Baier GP, Toussaint B, Jost B (2007). Anterior and posterior instability of the long head of the biceps tendon in rotator cuff tears: a new classification based on arthroscopic observations. Arthroscopy.

[6] Kang Y, Lee JW, Ahn JM, Lee E, Kang HS (2017). Instability of the long head of the biceps tendon in patients with rotator cuff tear: evaluation on magnetic resonance arthrography of the shoulder with arthroscopic correlation. Skeletal Radiol.

[7] Chae SH, Jung TW, Lee SH, Kim MJ, Park SM, Jung JY, Yoo JC (2020). Hidden long head of the biceps tendon instability and concealed intratendinous subscapularis tears. Orthop J Sports Med.

[8] Khil EK, Cha JG, Yi JS, Kim HJ, Min KD, Yoon YC, Jeon CH (2017). Detour sign in the diagnosis of subluxation of the long head of the biceps tendon with arthroscopic correlation. Br J Radiol.

[9] Varacallo MA, Seaman TJ, Mair SD (2024). Biceps Tendon Dislocation and Instability. StatPearls [Internet.

[10] Diplock B, Hing W, Marks D (2023). The long head of biceps at the shoulder: a scoping review. BMC Musculoskelet Disord.

[11] Sahin T (2020). Arm wrestling related injuries: a literature review. Int Arch Orthop Surg.

[12] Moloney DP, Feeley I, Hughes AJ, Merghani K, Sheehan E, Kennedy M (2021). Injuries associated with arm wrestling: a narrative review. J Clin Orthop Trauma.

[13] Şahbat Y, Kütük E, Çat G (2023). An unusual injury pattern: arm wrestling injury, treatment modalities, clinical outcomes, and return to sport. Ulus Travma Acil Cerrahi Derg.

